# Differences in the frequency of macrophage and T cell markers between focal and crescentic classes of anti-neutrophil cytoplasmic antibody (ANCA)-associated glomerulonephritis

**DOI:** 10.15171/jnp.2017.16

**Published:** 2016-12-25

**Authors:** Dana Kidder, Susan E Bray, Stewart Fleming

**Affiliations:** ^1^Renal Unit, Aberdeen, Royal Infirmary, Aberdeen, Scotland, AB25 2ZN; ^2^Tayside Tissue Bank, University of Dundee Medical School, Ninewells Hospital, Dundee, Scotland, DD1 9SY; ^3^Department of Pathology, University of Dundee Medical School, Ninewells Hospital, Dundee, Scotland, DD1 9SY

**Keywords:** Anti-neutrophil cytoplasmic antibody, Glomerulonephritis, Macrophage, T lymphocyte

## Abstract

**Background:**

Anti-neutrophil cytoplasmic antibody (ANCA)-associated glomerulonephritis (AAGN) can be classified into; focal, crescentic, mixed and sclerotic classes. Macrophages and T lymphocytes are key players in mediating renal injury. The frequency of macrophage and T lymphocytes in different histological classes is unclear.

**Objectives:**

We examined the frequency of macrophage and T lymphocyte markers in AAGN and assessed their correlation with renal function at presentation.

**Patients and Methods:**

Renal biopsies from 38 patients were included in immunohistochemistry analysis of macrophages (CD68, sialoadhesin [Sn] and mannose receptor [MR]) and T cells (CD4 and CD8) markers. The frequency of these markers in glomerular, periglomerular and interstitial compartments were measured in a blinded fashion. Biopsies were allocated a histological class of focal, crescentic, mixed or sclerotic. Scores were then matched to histological class and assessed for correlation with renal function.

**Results:**

The biopsies were crescentic 19 (50%), focal 10 (26.3%), mixed 6 (15.7%) and sclerotic 3 (8%). Interstitial CD68+ macrophages and CD8+ T lymphocytes showed best correlation with renal function at the time of presentation. CD68+ macrophages were significantly increased in crescentic compared to focal AAGN. MR+ macrophages, CD4 and CD8 T cells were also elevated in the interstitium of crescentic compared to focal group.

**Conclusions:**

In this study interstitial CD68 and CD8 showed the highest association with the renal function at presentation. Differences in the cellular infiltrate between focal and crescentic AAGN were related to CD68+ macrophages and to interstitial MR+ macrophages and T lymphocytes. Further studies are needed to assess these differences across all four histological categories.

**Implication for health policy/practice/research/medical education:**

This study highlights that in AAGN, the frequency of interstitial CD68+ macrophages and CD8+ lymphocytes show the highest correlation with renal function at presentation. Differences in the cellular infiltrate between focal and crescentic classes of AAGN are primarily related to CD68+ macrophages.

## Background

Anti-neutrophil cytoplasmic antibody (ANCA)-associated vasculitis (AAV) represents a group of inflammatory diseases characterized by multi-organ dysfunction. The disease can be divided into three categories based on clinical phenotype and ANCA types. These include granulomatosis with polyangiitis (formerly called Wegener’s granulomatosis), microscopic polyangiitis (MPA) and eosinophilic granulomatosis with polyangiitis (EGPA, formerly called Churg Strauss Vasculitis) (1). Kidney involvement is common, especially in GPA and MPA, and bears a significant impact on outcomes. Recently, an international working group developed a classification of renal involvement in into four different types based on glomerular pathology (2). ANCA-associated glomerulonephritis (AAGN) severity was categorised based on standard histological classification into crescentic (≥50% cellular crescents), focal (≥50% normal glomeruli), sclerotic (≥50% globally sclerosed glomeruli) and mixed. The classification was validated and confirmed to be directly associated (in ascending order of focal, crescentic, mixed and sclerotic) with renal outcomes e.g. renal function and end stage renal disease at 1 and 5 years (2).

Macrophages and T lymphocytes are key players in orchestrating and inducing damage in AAGN (3-5). Sn (also known as Siglec-1, CD169) is the prototype of the family of sialic acid binding immunoglobulin-like lectins (Siglecs). Sn+ macrophages are found in the marginal zone of the spleen and subcapsular sinus of lymph nodes. However, Sn expression can be induced under inflammatory conditions, such as: proliferative glomerulonephritis and rheumatoid arthritis (6). The frequency of Sn+ macrophages was found to be correlated directly to the severity of renal injury in a murine model of systemic lupus erythematosus (7). Mannose receptor (MR) is a pattern recognition receptor implicated in the uptake of endogenous and microbial ligands. It is up-regulated on alternatively activated macrophages and is involved in antigen presentation to T cells (8). MR deficiency was found to be protective in an animal model of crescentic glomerulonephritis (9).

## 2. Objectives

The aim of this study was to examine the correlation between specific macrophage and T cell markers and renal function at the time of presentation with AAGN. The second aim of this study was to assess for differences in the cellular infiltrate between focal and crescentic AAGN.

## 3. Patients and Methods

### 3.1. Study population

The pathology database in Ninewells Hospital, Dundee, Scotland was used to search for all patients with AAV who had a renal biopsy between 2003-2012. Sample identification and subsequent slide development and staining were performed by Tayside Tissue Bank (TTB) following Tenovus Scotland Grant award (Grant number T13/15) and Research Ethics Committee Approval. Forty-five cases were identified by electronic search and out of these only 38 cases had sufficient renal tissues available for analysis in this study. All biopsies were of patients with their first presentation of AAV with suspected renal involvement. Sample retrieval, processing and optimising staining were performed by SEB. The markers used in the analysis included CD68, Sn, MR, CD4 and CD8. Morphologically normal kidney specimens from 20 individual nephrectomies were used as controls.

### 3.2. Immunohistochemistry

Antigen retrieval and deparaffinization was performed using DAKO EnVision™ FLEX Target Retrieval solution (high pH) buffer in a DAKO PT Link. Immunostaining using DAKO EnVision™ FLEX system on a DAKO Autostainer Link48 was carried out according to manufacturer’s protocol. Sections were incubated with primary antibody for 30 minutes. The following primary antibodies were used: anti-CD68 (clone PG-M1, DAKO, dilution 1:100), anti-Sn (HPA053457, Atlas Antibodies, dilution 1:200), anti-MR (Ab64693, Abcam, dilution 1:2000), anti-CD4 (clone 4B14, Leica Biosystems, dilution 1:25) and anti-CD8 (clone C8/144B, DAKO, dilution 1:50) antibodies. DAKO substrate working solution was used as a chromogenic agent for 2 × 5 minutes and sections were counterstained in EnVision™ FLEX haematoxylin. Sections known to stain positively were included in each batch and negative controls were prepared by replacing the primary antibody with DAKO antibody diluent.

Slide scoring was performed by DK in a blinded fashion. The frequency of cellular markers in the glomerular, peri-glomerular and interstitial compartments were examined. The mean of the cellular markers for the total number of glomeruli examined was used as a final glomerular score. A similar approach was used for peri-glomerular infiltrate. For interstitial scores, the frequency of the cellular markers in 8 high power microscopic fields (×400) was calculated and the mean score was used. Histological severity grading was performed by SF in a blinded fashion. The immunohistochemistry and histological severity were matched following de anonymising of the data.

### 3.3. Ethical issues

The research followed the tenets of the Declaration of Helsinki. The research was approved by ethical committee of University of Dundee and NHS Tayside.

### 3.4 Statistical analysis

Data analysis was performed with GraphPad Prism version 4. One-way analysis of variance (ANOVA) with Kruskal-Wallis test was used in comparing cellular markers in histological severity groups. Mann-Whitney U was used to compare crescentic and focal groups. *P* values of <0.05 was considered significant.

## 4. Results

### 4.1. Patients characteristics

Renal biopsies from 38 patients with ANCA-associated renal vasculitis were examined. All patients were Caucasian. The median age at the time of presentation was 66 years (range 25-76). 24 (63%) had MPA and 14 (37%) had diagnosis of GPA. 37 (97%) of patients were ANCA positive (62% MPO+ and 38% PR3+). The median serum creatinine at presentation was 297 µmol/L (Table 1). The distribution of the histological classification was as follows: crescentic 19 (50%), focal 10 (26.3%), mixed 6 (15.7%) and sclerotic 3 (8%).

**Table 1:** Patients characteristics at presentation

	**All biopsies**	**Focal**	**Crescentic**	**Mixed**	**Sclerotic**
Patient number (%)	38	10 (26.3)	19 (50)	6 (15.7)	3 (8)
Age at biopsy (range)	66 (25-76)	64 (53-85)	65 (55-79)	67 (49.5-73)	66
Sex (M/F)	19/19	4/6	12/7	1/5	2/1
Number of glomeruli (range)	15 (9-24)	19 (8-31)	13 (8-16)	22 (13-26)	24
PR3+/MPO+	14/23	3/7	8/11	2/4	1/1
Serum creatinine µmol/L (range)	297 (210-484)	273 (99-489)	294 (213-422)	292 (162-475)	989
eGFR mL/min/1.73 m^2^(range)	18 (10-26)	19 (12-55)	10 (10-28)	16 (11-35)	5

### 4.2. Correlation between histological markers and renal function

The frequency of macrophage (CD68, Sn and MR) and T cells (CD4 and CD8) markers were examined in the glomerular, peri-glomerular and interstitial compartment and compared to renal function at the time of presentation as measured by eGFR. Interstitial CD68+ macrophages and CD8+ T lymphocytes had a significant negative correlation with eGFR (Table 2). No significant correlation was observed between the frequency of Sn+ macrophages, MR+ macrophages or CD4+ T cells and eGFR. There were no significant differences in the frequency of all markers based on the clinical diagnosis of GPA versus MPA (data not shown).

**Table 2:** The frequency of individual markers in different renal compartments

**Marker/compartment**	**Focal** **(a)**	**Crescentic (b)**	**Mixed** **(c)**	**Sclerotic (d)**	**Control** **(e)**	**Intergroups comparison**	**Correlation with eGFR,** **Spearman r (P value)**
**CD68**							
Glomerular	11 ± 3.43	17.4 ± 2.16	14.9 ± 4.1	4.2 ± 0.44	2 ± 0.21	a-b*, b-e***, c-e**	-0.05 (0.7)
Periglomerular	8.6 ± 1.07	16.3 ± 1.85	13.25 ± 2.75	11.3 ± 2.9	3.4 ± 0.6	a-b*, b-e***, c-e**	-0.24(0.1)
Interstitial	39 ± 5.9	119 ± 14.1	86.7 ± 16.7	116 ± 31.4	11.6 ± 1.5	a-b*, b-e***, c-e *, d-e*	-0.44 (0.007) **
**Sn**							
Glomerular	0.7 ± 0.5	0.5 ± 0.2	1.3 ± 0.7	0	0	NS	-0.12 (0.4)
Periglomerular	2.3 ± 1.3	2.6 ± 0.8	5.9 ± 2	0.5 ± 0.26	0	b-e*, c-e**	-0.23 (0.1)
Interstitial	17 ± 10.1	24.2 ± 5.2	28.1 ± 5.1	23.8 ± 8	0.8 ± 0.3	b-e***, c-e**	-0.06 (0.7)
**MR**							
Glomerular	1.6 ± 0.6	4.6 ± 1.2	4 ± 0.9	2.7 ± 0.4	7.6 ± 0.6	a-e***	-0.11 (0.5)
Periglomerular	5.1 ± 1.3	9.1 ± 1.3	10 ± 1.2	6.1 ± 1.3	1.5 ± 0.2	b-e***, c-e**	-0.11 (0.5)
Interstitial	17.6 ± 5.5	33.6 ± 4	25.7 ± 8.9	27.7 ± 6.1	2.5 ± 0.3	a-b*, b-e***, c-e*, d-e*	-0.30 (0.08)
**CD8**							
Glomerular	0.5 ± 0.32	2.07 ± 0.64	1.65 ± 1	1.47 ± 0.8	0.4 ± 0.11	NS	0.11 (0.5)
Periglomerular	6 ± 1.7	9 ± 1.5	8.3 ± 3.6	10.1 ± 7	0.08 ± 0.06	b-e**	-0.26 (0.1)
Interstitial	20.4 ± 3.8	43.3 ± 4.6	37 ± 6.8	54 ± 18.3	2.2 ± 0.4	a-b**, a-c*, b-e***, c-e*, d-e**	-0.34 (0.04)*
**CD4**							
Glomerular	3.4 ± 1.2	1.7 ± 1.4	1.3 ± 0.45	0.03 ± 0.03	0.7 ± 0.1	NS	0.06 (0.7)
Periglomerular	3.8 ± 1.7	9.1 ± 2.1	10.2 ± 4.6	5.1 ± 4.4	0	b-e**	-0.06 (0.7)
Interstitial	13.6 ± 3.8	36.9 ± 6	25 ± 4.4	50.7 ± 9.5	3 ± 0.6	a-b*, b-e **, d-e**	-0.32 (0.05)

NS= no significant differences. P value *≤0.05, **≤0.01 and ***<0.001.

### 4.3. Differences between focal and crescentic classes

**Figure 1:**
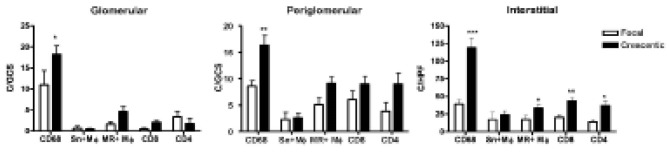
The distribution of macrophage and T cell markers in glomerular, periglomerular and interstitial compartments in focal and crescentic groups.

We examined the frequency of the cellular markers in different histological classes and control tissues. Higher glomerular, peri-glomerular and interstitial CD68+ macrophages were found in the crescentic compared to focal class (Table 2 and Figure 1). No significant differences in glomerular or peri-glomerular Sn+ macrophages, MR+ macrophages, CD4+ or CD8+ T lymphocytes were observed between focal and crescentic groups. In the interstitial compartment, significant increase in the frequency of MR+ macrophages, CD4+ and CD8+ T cells was seen in the crescentic compared to focal class (Figure 1).

In the mixed group, the frequency of CD68+ macrophages, MR+ macrophages, CD8 and CD4 T cells, were non-significantly lower than crescentic and higher than focal groups (Table 2). Sn+ macrophages were non-significantly higher in the mixed group (peri-glomerular and interstitial). The sclerotic group showed a significantly elevated frequency of interstitial CD68+ macrophages, MR+ macrophages and CD8 T lymphocytes compared to controls. Sn+ macrophages were present in peri-glomerular (crescentic and mixed groups) and interstitial (crescentic, mixed and sclerotic) compartments (Table 2).

## 5. Discussion

Previous studies on pathogenesis of AAGN revealed significant contribution of cell mediated immunity to the renal and tissue injury (3-5,10). In this study we examined the frequency of macrophage (CD68, Sn and MR) and T lymphocytes (CD8 and CD4) markers in renal biopsies of different classes of AAGN. We found that the frequency of interstitial CD68+ macrophages, followed by interstitial CD8+ T lymphocytes correlated best with the renal function at the time of presentation. We also identified the presence of significant differences in CD68+ macrophages in glomerular, periglomerular and interstitial compartments between focal and crescentic classes. Further comparison between these two classes revealed higher frequency of MR+ macrophages, CD8 and CD4 T lymphocytes only in the interstitial compartment.

Similar to previous studies, we showed that the only macrophage marker that consistently correlated with renal impairment and class differences was CD68 (panmacrophage marker). Despite a wealth of evidence implicating macrophages in the pathogenesis of AAV glomerulonephritis, the phenotype of these macrophages remain elusive (3-5,10,11). Ikezumi et al, found that Sn+ macrophages, a subset of CD68+ macrophages were correlated with degree of proteinuria and interstitial damage in proliferative GN (12). In our study, Sn+ macrophages were predominantly observed in the interstitial compartment, did not significantly differ between histological classes and were not an association with renal function at the time of presentation. Sn+ macrophages were present in high frequency in the sclerotic group, albeit with limited number of samples. It remains unclear whether this subset of macrophages might play a role in fibrosis. Previously, MR deficient mice were shown to be protected from experimental nephritis that was associated with a shift towards an anti-inflammatory macrophage phenotype (6). The evidence for the role MR in human AAGN is unknown. In our study, higher interstitial MR+ macrophages were found in crescentic as compared with focal AAGN. Similar to Sn, MR did not show a significant correlation with renal function at the time of presentation. These data suggest that although the severity of tissue injury in AAGN is directly related to the overall macrophage burden and the relative contribution macrophage subsets is not clear. Recently an elegant study of MPO+ AAGN showed that MPO+ macrophages were strongly associated with tissue injury and renal impairment (13). However, the phenotype of these macrophages remains unclear.

T cells have been shown in both animal models of AAV GN and human AAV to be key players in mediating inflammation (14). Our analysis is in agreement with previous studies showing significant correlation between CD8+ T cells and degree of renal impairment. We extend these observations further by the finding of higher frequency of CD8+ T cells in the interstitium in a graded fashion between focal, crescentic and mixed classes. This observation not only emphasize their role in predicting outcomes (15) but also stress on the complementary role of interstitium in addition to the glomerular compartment in defining outcomes following AAV GN.

## 6. Conclusions

In conclusion, this study showed that among the markers studies, CD68 and CD8 showed the highest association with the renal function at the time of presentation. Differences in the cellular infiltrate between focal and crescentic AAGN were primarily related to CD68 and to a lesser extent with interstitial MR+ macrophages and T lymphocytes. Interstitial rather than glomerular or peri-glomerular cellular infiltrate correlated better with renal function at presentation. Further studies including larger number of biopsy samples are needed to asses for differences across all four histological categories of AAV.

## Limitations of the study

The main limitation of this study is the relatively small sample size. A larger sample size with more representation in the mixed and sclerotic groups would have allowed for more robust comparison between all classes. As the study was done in a blinded fashion, the distribution of cases was not even across histological classes with majority of cases being under crescentic and focal categories. Another limitation of this study is the absence of neutrophil and B cell markers to compare their frequencies in different compartments with that of macrophages and T cells.

## Authors’ contribution

DK and SF designed the study. SEB performed immunohistochemistry. DK scored and analyzed immunohistochemistry slides. SF reviewed and scored histological classes. DK, SEB and SF participated in writing the paper.

## Conflicts of interest

The authors declare no conflict of interest.

## Funding/Support

This study was supported by Tenovus Scotland/Tayside grant award to DK (Grant # T13/15).
